# CD277 is a Negative Co-stimulatory Molecule Universally Expressed by Ovarian Cancer Microenvironmental Cells

**DOI:** 10.18632/oncotarget.165

**Published:** 2010-09-11

**Authors:** Juan R. Cubillos-Ruiz, Diana Martinez, Uciane K. Scarlett, Melanie R. Rutkowski, Yolanda C. Nesbeth, Ana L. Camposeco-Jacobs, Jose R. Conejo-Garcia

**Affiliations:** ^1^Department of Microbiology and Immunology, Dartmouth Medical School, Lebanon, NH 03756, USA; ^2^Department of Medicine, Dartmouth Medical School, Lebanon, NH 03756, USA

**Keywords:** Tumor microenvironment, tumor immunology, dendritic cell, immune evasion, butyrophilin, immunotherapy

## Abstract

CD277, a member of the butyrophilin subfamily 3 (BTN3), shares significant sequence similarities and predicted common structural features with inhibitory B7-H4 and other members of the B7 superfamily. Here we report that CD277 is consistently expressed in stromal, as well as tumor cells in the microenvironment of human advanced ovarian carcinoma specimens, both of primary and metastatic origin. MHC-II^+^ myeloid antigenpresenting leukocytes (dendritic cells and macrophages) express significantly higher levels of surface CD277, compared to other tumor-infiltrating leukocyte subsets, and this expression is significantly up-regulated by multiple common tumor microenvironmental signals, including VEGF and CCL3. Most importantly, engagement of CD277 on the surface of TCR-stimulated T cells inhibits their otherwise robust expansion and production of Th1 cytokines by preventing the up-regulation of cFLIP. Our results point to a role for CD277 up-regulated by microenvironmental signals in the acquisition of a regulatory phenotype by tumor-associated myeloid cells. Consequently, CD277, and likely other butyrophilins and butyrophilin-like molecules, emerge as regular players in the orchestration of immunosuppressive networks in ovarian cancer, and therefore new targets for interventions to overcome immune evasion and boost anti-tumor immunity in cancer patients.

## INTRODUCTION

Despite being an underfunded disease, epithelial ovarian cancer is responsible for the death of ~15,000 Americans per year, even more than melanoma or brain tumors[1]. Five-year survival rates have improved little in the last 30 years, and still remain at 30%, at best, for patients with metastatic ovarian carcinoma, the stage at which most cases are diagnosed[1, 2]. The standard course of treatment for ovarian cancer, surgical debulking followed by chemotherapy, usually eliminates detectable tumors and puts the cancer into remission. Unfortunately, most patients relapse within 2 years and the tumors that then develop are commonly resistant to chemotherapy and are frequently fatal. We and others have demonstrated that in the ovarian carcinoma microenvironment only T cells can spontaneously exert clinically relevant pressure against tumor progression. Simply defining the location where tumor-infiltrating T lymphocytes accumulate predicts the patient's outcome[3-7]. However, as the dismal statistics show, spontaneously developed antitumor immunity is too often insufficient. In part, this is because many cancers, including ovarian cancer[8-15], suppress the antitumor immune response by expressing molecules that induce immune tolerance to the tumor by recruiting immunosuppressive stromal leukocytes in the tumor microenvironment (as elegantly reviewed by W. Zou[14]). One area of great potential is stimulating antitumor immunity, but immunological understanding of the epithelial ovarian cancer tumor microenvironment is limited[8-10, 16-18], and tumor-induced immunosuppression has so far impeded the development of improved immunotherapies. The particularly immunosuppressive nature of the ovarian carcinoma microenvironment has even abrogated the effectiveness of the most promising strategies based on stimulating T cell-mediated immune responses through adoptive transfer[10, 16, 19]. A better understanding of the peculiarities of this particularly abrasive microenvironment is therefore urgently required.

Engagement of co-inhibitory molecules plays a crucial role in the abrogation of T cell responses in the context of cancer and chronic infections[20]. Signals mediated by members of the B7 family of molecules, including PD-L1 and B7-H4, appear to be crucial for the orchestration of an immunosuppressive microenvironment in cancer patients in general[15, 21, 22], and both are abundantly expressed in ovarian tumors in particular[12, 15, 22, 23].

Interestingly, butyrophilin (BTNs) and butyrophilin-like molecules share significant sequence homology with B7 family members, but little is known about their functions in specific biological contexts. BTNs are transmembrane proteins belonging to the Ig superfamily that can be divided into three groups, termed BTN1, BTN2 and BTN3.

Emerging results indicate that some BTN1 and BTN2 molecules could play a role in the impairment of T cell responses[24]. In addition, BTNL2, a BTN/B7-like molecule has been identified as a negative co-stimulatory molecule modulated in intestinal inflammation and also associated with sarcoidosis[25-27]. However, little is known regarding the potential of BTN3 subfamily members as immunosuppressive ligands. The role of BTNs in the modulation of spontaneous anti-tumor immune responses has never been investigated, despite the fact that BTNs are expressed on a variety of immune cells and tumor cell lines in a pattern identical to that of co-inhibitory molecules of the B7 family, and could thus play a similar tolerogenic role[28].

Here we report that CD277, a member of the butyrophilin subfamily 3 (BTN3), is universally expressed by myeloid APCs and tumor cells in the human ovarian carcinoma microenvironment and inhibits the TCR-mediated expansion of human T cells as well as the production of Th1 cytokines. Our results point to CD277 as a new target for the design of novel interventions to overcome immune evasion and boost anti-tumor immunity in ovarian cancer patients.

## RESULTS

### CD277 shares significant sequence similarities and common structural features with inhibitory B7-H4

**Figure 1:**
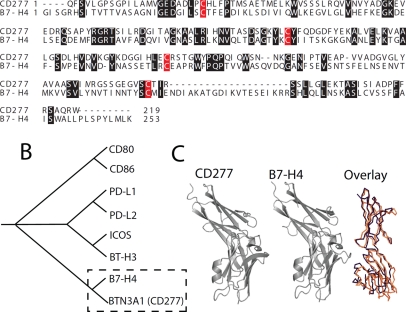
CD277 shares sequence and structural similarity with other members of the B7 superfamily of costimulatory molecules (A) Alignment of CD277 and B7-H4. Conserved cysteine residues are highlighted in red. Conserved Amino Acid regions are highlighted in black. (B) Cluster analysis of B7 superfamily members. The Amino Acid sequence of CD277 was aligned to other B7 superfamily members and a similarity tree was generated. (C) (left and center) Presentation of the CD277 and B7-H4 structures, predicted using the know structure of PD-L1 as a model, based on sequence. (right) Schematic overlay of both predicted structures. Blue, CD277; Orange, B7-H4.

B7-H4 plays a crucial role in the orchestration of immune evasion in the microenvironment of human ovarian carcinomas, where it is primarily expressed on the surface of tumor-associated macrophages and tumor cells, and interacts with an unidentified receptor in T cells[15, 22]. We identified that the extracellular domain of CD277/BTN3A1, a type I transmembrane member of the butyrophilin family, shows a striking similarity with the extracellular domain of immunosuppressive B7-H4, with 26% identities including a conserved pattern of 4 cysteins in identical positions (Figure 1A). Similar to B7-H4, CD277 is expressed on leukocytes including antigen-presenting cells[28]. Although CD277 shows a high degree of resemblance with other members of the B7 superfamily (not shown), it clusters more closely with B7-H4 when a similarity tree is generated based on amino acid sequence alignment (Figure 1B). Correspondingly, the predicted three-dimensional structure of the extracellular domains of both molecules revealed multiple common features and the presence of a similar overall tertiary structure (Figure 1C).

### CD277 inhibits the TCR-mediated proliferation of activated human T cells

**Figure 2:**
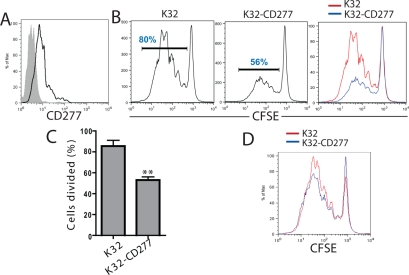
CD277 inhibits TCR-mediated human T cell proliferation (A) FACS analysis of CD277 expression by mock-transduced K32 (aAPCs) cells (solid) and K32 cells stably infected with a retroviral construct encoding CD277 (open). (B) Untransduced or CD277-expressing K32 cells were loaded with anti-CD3 agonistic antibodies (OKT3; 100 ng/ml) and cells were then co-cultured (1:10) with CFSE-labeled human peripheral blood T cells. The percentage of proliferating T cells was analyzed by FACS after five days of proliferation. Data are representative of five independent experiments with similar results. (C) Representative FACS analysis of the data shown in B. **P<0.05 (Mann-Whitney). (D) CD28 costimulation alleviates CD277-mediated suppression. K32 cells were loaded with anti-CD3 and anti-CD28 agonistic antibodies (25 ng) and cells were cocultured with human T cells, as described above. CFSE dilution was evaluated by FACS five days later. Data are representative of four independent experiments with similar results

Based on the sequence and structural similarity of CD277 and B7-H4, we hypothesized that CD277 could also act as a negative co-stimulatory molecule that regulates T cell activation. To define the effect of increased CD277 availability on T cell proliferation, we used an established system to induce the expansion of human T cells based on artificial antigen-presenting cells (aAPCs)[29, 30]. Briefly, K562 cells, which lack MHC-I expression, were transduced with CD32 to generate K32 cells. K32 cells were irradiated, coated with different concentrations of activating CD3 and CD28 antibodies and incubated with naïve T cells, to induce rapid proliferation. We transduced these aAPCs with the open-reading frame of CD277 (NM_007048) and confirmed the presence of the protein on their surface by FACS analysis (Figure 2A).

As shown in Figure 2B, the TCR-induced proliferation of naïve human T cells was significantly impaired when CD77 was expressed on aAPCs in 5 independent experiments (30% decrease in total proliferating cells, Figure 2C; 78 % decrease in the division index, not shown). Of note, impairing the strong proliferative activity elicited by optimal concentrations of agonistic anti-CD3 antibodies (100 ng/ml) on human T cells, implies a profound inhibitory effect. Importantly, the immunosuppressive effect of CD277 could be alleviated, but not completely restored, in the presence of CD28 co-stimulation (Figure 2D). In contrast, K32 aAPCs transduced with the leukocyte ligand PILAR[30] dramatically enhanced T cell proliferation under identical experimental conditions (data not shown).

**Figure 3:**
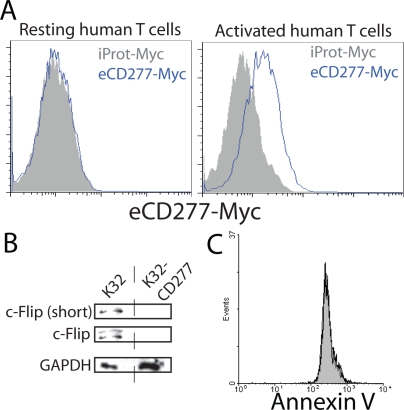
CD277 inhibits cFLIP in activated human T cells (A) Resting human T cells or T cells activated for 24 hours with anti-CD3/anti-CD28 beads were incubated with a Myc-tagged irrelevant protein or a with Myc-tagged extracellular form of CD277. Protein binding to the cell surface was analyzed by FACS using a secondary anti-Myc antibody. Data are representative of three independent experiments with similar results. iProt, irrelevant protein. eCD277, extracellular region of CD277. (B) Human T cells were cocultured for 24 hours with K32 cells ectopically expressing or not CD277 and whole cell lysates were used to analyze c-Flip expression by Western blot. Data are representative of two independent experiments with similar results. (C) Resting human T cells were activated for 24 hours with K32 cells ectopically expressing (solid) or not (open) CD277 and intracellular expression of Annexin V was determined.

Most importantly, the regulatory effect of CD277 appears to be mediated by a receptor selectively expressed on the surface of activated T cells, because only CD3/CD28-stimulated, but not naïve T cells, bound to a Myc-tagged recombinant extracellular domain of CD277 (Figure 3A).

Together, these data suggest that CD277, similar to various members of the B7 superfamily of co-inhibitory molecules, plays a role in fine tuning of antigen-specific responses by binding to an inhibitory receptor expressed on the surface of activated T cells. These results also suggest that CD277 may participate in the balance between activation and inhibition during T cell homeostasis.

### CD277 inhibits cytokine secretion and c-Flip expression by human T cells

TCR-mediated activation of T cells results in increased expression of cellular caspase-8 (FLICE)-like inhibitory protein (cFLIP), which augments caspase-8 activity, necessary to initiate the activation of nuclear factor-B (NF-B) and promote T cell proliferation[31]. We therefore compared cFLIP expression on T cells stimulated with agonistic CD3 antibodies in the presence or the absence of CD277. As shown in Figure 3B, increasing CD277 availability completely abrogated the expression of c-FLIP after 24 h of stimulation. Interestingly, impairment of T cell expansion by CD277 expression was not caused by a direct apoptotic effect, because intracellular staining of Annexin V produced identical signals in T cells expanded in the presence or the absence of ectopic CD277 in multiple experiments (Figure 3C).

**Figure 4:**
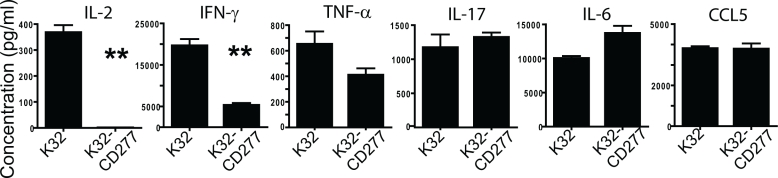
CD277 inhibits Th1 cytokine secretion by TCR-stimulated human T cells K32 cells expressing or not CD277 were loaded with anti-CD3 agonistic antibodies and cells were cocultured with human T cells as described above. Culture supernatants were collected after five days and cytokine secretion was analyzed by Multiplex analysis. Data are representative of two independent experiments with similar results. **P<0.05 (Mann-Whitney).

Importantly, CD277 over-expression profoundly decreased the production of Th1 cytokines IL-2 and INF-g by stimulated T cells, although the production of TNF-a was only slightly affected on a per cell basis (Figure 4). In contrast, IL-17 and, especially, IL-6 production was enhanced in the presence of CD277.

Together, these data indicate that CD277 impairs the expansion of TCR-stimulated T cells by preventing the up-regulation of cFLIP, which results in a dramatic decrease in Th1 cytokine production.

### CD277 is abundantly expressed in all advanced human ovarian carcinoma specimens analyzed

**Figure 5:**
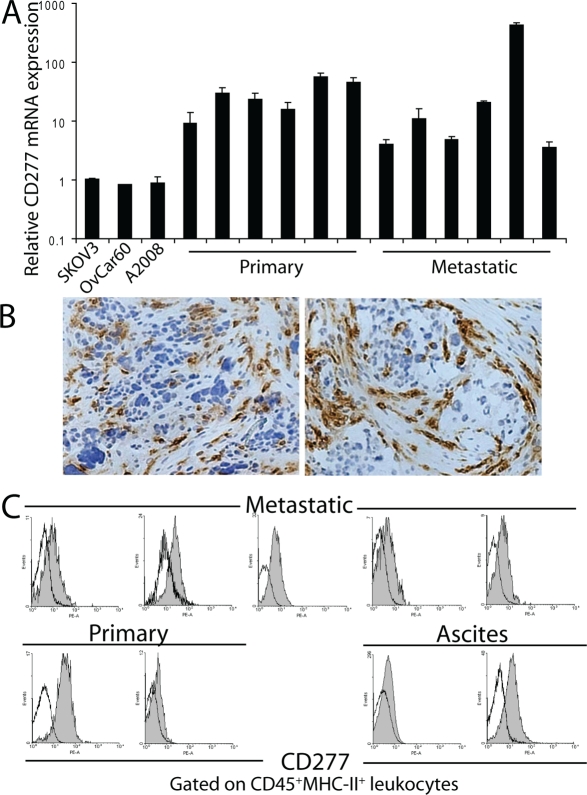
CD277 is abundantly expressed in the microenvironment of human epithelial ovarian cancer (A) Real Time RT-PCR analysis of CD277 mRNA levels in human ovarian cancer cells lines (SKOV3, Ovcar60 and A2008) and multiple human tumor specimens. (B) Representative immunohistochemistry of CD277 protein expression in ovarian tumors from two different patients (magnification, x200). (C) Single-cell suspensions from different stage III-IV human ovarian tumors and ascites were procured and surface expression of CD277 on CD45+MHC-II+ leukocytes (CD20-)[13] was determined by FACS. Open histograms represent staining with isotype control antibodies, solid histograms indicate CD277 staining.

Having demonstrated that CD277 acts as a negative signal to modulate T cell proliferation we hypothesized that, similar to structurally related B7-H4, CD277 could also play a role in immune evasion in the ovarian carcinoma microenvironment. To test this hypothesis, we first confirmed that 12 unselected stage III/IV human ovarian carcinoma solid specimens from our tumor bank expressed significant mRNA levels of *CD277* (Figure 5A). Comparable levels of inhibitory *CD277* were detected in samples from the primary tumor (n=6) and metastatic masses (n=6). In addition, 3 out of 3 established ovarian cancer cell lines analyzed also express detectable *CD277* (Figure 5A). Correspondingly, immunohistochemical analysis of 30 tumor specimens from patients with epithelial ovarian carcinoma (including both metastatic and primary masses) revealed that all specimens analyzed were strongly positive for CD277 protein (Figure 5B), while no positive signal was detected with the isotype control antibody. CD277 signal was found in abundant cells in the stroma as well as tumor islets (Figure 5B, left). In several histological sections, a layer of non-tumor CD277+ cells distributed in a vascular-like pattern was found around tumor islets (Figure 5B, right). No differences between primary vs. metastatic specimens were noted.

These data suggest that inhibition of T cell-mediated anti-tumor immune responses by immunosuppressive CD277 may be a common mechanism of evasion orchestrated by stromal and tumor cells in the microenvironment of advanced human epithelial ovarian cancers.

### CD277 is expressed by human ovarian cancer microenvironmental antigen-presenting cells

To define the precise cell types expressing immunosuppressive CD277 in the human ovarian carcinoma microenvironment, we mechanically dissociated 7 randomly received fresh stage III/IV epithelial ovarian cancer samples, which included 2 primary and 5 metastatic specimens. FACS analysis of these freshly prepared single cell suspensions revealed that CD277 was most highly expressed on the surface of CD45+ MHC-II+ leukocytes in all samples (Figure 5C). Although the precise categorization of these leukocytes is complicated by the fact that they co-express other macrophage and myeloid-derived suppressor cell (MDSC) markers, we have previously demonstrated that in the solid tumor microenvironment in humans most of these leukocytes express low but detectable levels of phenotypic markers of immature but bona fide dendritic cells (DCs), including CD11c, DEC205 and CD86, and are negative for CD20 and therefore not B cells[8-10, 13, 18]. We initially termed these cells as Vascular Leukocytes (VLCs) because they up-regulate endothelial markers at perivascular locations in tumors. Thus, the distribution of CD277+ structures around tumor islets found in some specimens is consistent with the perivascular homing of VLCs in ovarian cancer[32, 33]. In addition, CD45+MHC-II+ leukocytes in tumor ascites (primarily canonical macrophages in our hands) also expressed significant levels of surface CD277 (Figure 5C).

**Figure 6:**
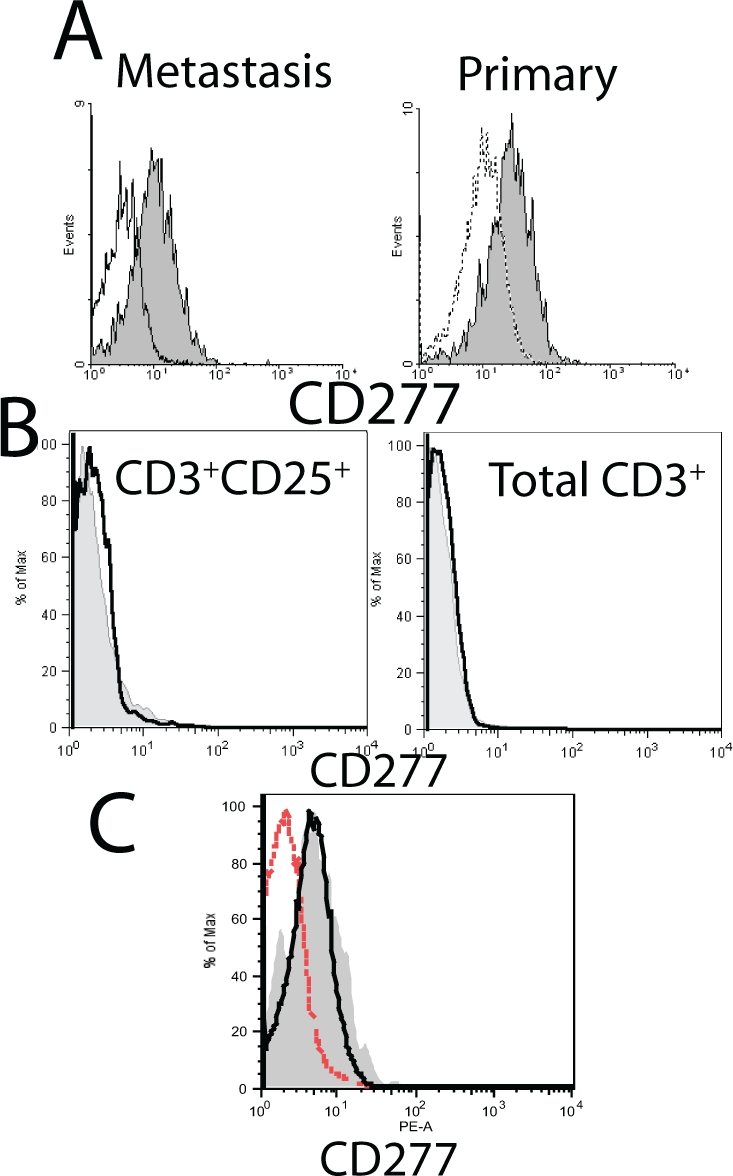
CD277 is preferentially expressed by APCs and tumor cells in the ovarian carcinoma microenvironment (A) Representative FACS analysis of CD277 expression on CD45+MHC-II+CD20- (solid), compared to that of other CD45+ cells (open) within the same human specimens. (B) FACS analysis of CD277 on CD4+CD25+ (regulatory + activated T cells) and total CD3+ T cells in a metastatic ovarian cancer tumor. Solid histograms indicate CD277 expression, open histograms represent isotype control staining. Identical results were obtained when primary tumors or ascites were analyzed for CD277 expression on T cells. (C) Representative FACS analysis of CD277 expression on CD45- cells in dissociated ovarian cancer specimens (mainly tumor cells; open thicked), compared to that in CD45+MHC-II+CD20- inflammatory leukocytes (solid) in the same specimen. Open dashed histograms represent isotype control staining. Comparable results were obtained in primary and metastatic tumors.

Expression of CD277 in tumor-associated MHC-II+ DC/macrophages was significantly higher than that in other ovarian cancer microenvironmental leukocyte subsets in most specimens analyzed, both primary and metastatic (Figure 6A). Interestingly, CD3+ T cells infiltrating ovarian carcinoma specimens, including regulatory T cells (CD3+CD4+CD25+) did not show detectable levels of CD277 (Figure 6B). In contrast, variable but substantial levels of CD277 were found in CD45- cells (Figure 6C) suggesting that, similar to established tumor cell lines, ovarian tumor cells also up-regulate CD277 as a mechanism of immune evasion.

Together, these data indicate that inhibitory CD277 is consistently up-regulated by abundant stromal and tumor cells in the ovarian cancer microenvironment, thus pointing to a crucial role for this molecule in the orchestration of immunosuppressive networks.

### CD277 expression is up-regulated in human DCs by inflammatory cytokines and hypoxia-associated mediators

**Figure 7:**
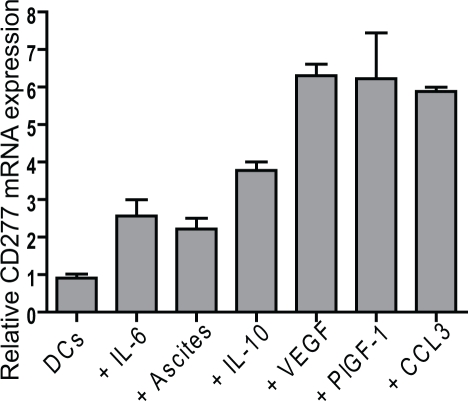
Tumor microenvironmental inflammatory cytokines and hypoxia-associated mediators up-regulate the expression of CD277 in monocyte-derived human DCs Monocyte-derived DCs were incubated for 18 hours with the indicated cytokines and CD277 expression was evaluated by Real Time Quantitative PCR.

Because CD277 is over-expressed in tumor microenvironmental MHC-II+ DC/macrophages, we finally aimed to define microenvironmental factors mediating its up-regulation. We therefore generated DCs from human aphaeresis samples and incubated them with a variety molecules frequently over-expressed in the tumor microenvironment, and determined how CD277 levels were impacted. As shown in Figure 7, DCs expressed low but detectable levels of CD277, which were further up-regulated upon incubation with tumor-derived ascites or inflammatory IL-6. Interestingly, hypoxia-induced VEGF and PlGF, commonly up-regulated in most solid tumors, induced the highest up-regulation of CD277 in human DCs. Among common inflammatory cytokines, incubation with CCL3 dramatically increased the expression of CD277 too. CCL3 is, interestingly, produced at high levels by tumor-associated DCs (unpublished observations), and therefore abundantly over-produced in the tumor microenvironment.

Together, these data indicate that multiple common tumor microenvironmental signals induce the up-regulation of CD277 in tumor-associated APCs, thus stimulating their acquisition of a regulatory phenotype.

## DISCUSION

Positive and negative co-stimulatory molecules cooperatively determine the activation of T cells[34]. A growing body of evidence indicates that multiple negative co-stimulatory molecules expressed by tumor and tumor microenvironmental cells are crucial to confer immune privilege against T cell-mediated eradication. Here we show that CD277, a member of the BTN family, inactivates T cell function and is consistently expressed by ovarian cancer-associated MHC-II+ DC/macrophages leukocytes as well as primary ovarian cancer cells in all specimens analyzed.

CD277 shares significant sequence identities and common structural attributes with inhibitory B7-H4. Similar to B7-H4 and also to other members of the B7 superfamily of co-inhibitory molecules such as PD-L1, CD277 impairs TCR-induced proliferation and Th1 cytokine production by human T cells. These inhibitory effects are associated with a blockade in the up-regulation of cFLIP, necessary to promote T cell proliferation[31]. Although impairment of T cell activation by other members of related sub-families of BTNs[24], or BTN-like molecules[25, 26], has been recently proposed, the role of CD277, or any other member of the BTN3 sub-family as a ligand, remained completely unknown. During the preparation of this manuscript, a report showing the effect of signaling directly through BTN3 in lymphocytes was published online[35]. The presence of CD277 on the surface of T cells has been confirmed by others[28], although our results indicate that in the ovarian microenvironment, little to no expression of CD277 can be detected on the surface of tumor-infiltrating lymphocytes, including regulatory T cells, using commercial antibodies. In contrast, we found that CD277 is significantly expressed in myeloid MHC-II+ leukocytes (primarily DCs, in our hands[8-10, 13, 18]) and tumor cells. Therefore, the inhibition of the anti-tumor function of T cells at tumor locations should be mediated by the engagement of CD277 expressed on APCs or tumor cells with another receptor expressed in T cells. In fact we confirmed that CD277 selectively binds to the surface of activated, but not resting T cells, indicating that the inhibitory activity of CD277 depends on the stimulation of a receptor that is expressed during T cell activation. Due to the structurally similar features of CD277 and PD-L1 (not shown), we surmised that PD-1, which is up-regulated in T cells upon activation, could mediate the observed non-apoptotic regulatory effects. However, PD-1 has been previously ruled out as a binding partner of CD277 by others[28], and we were not able to generate conclusive evidence for CD277:PD-1 interactions. The receptor for CD277, similar to the receptor for other BTNs or B7-H4, therefore remains to be identified.

Also similar to B7-H4 and PD-L1, CD277 is expressed by both leukocytes and non-hematopoietic tumor cells, suggesting that it may impair T cell function at the tissue level, beyond the lymph node where T cell priming takes place. Correspondingly, we found that CD277 is consistently expressed in all human advanced ovarian carcinoma specimens analyzed, and comparable levels and patterns of expression were found in metastatic and advanced (stage III/IV) primary tumor specimens. Indeed, we found that CD277 is up-regulated in monocyte-derived DCs by multiple inflammatory cytokines and hypoxia-induced mediators commonly found in the tumor microenvironment, including VEGF, and CCL3 and, to a lesser extent, IL-6. Our data indicate that both CCL3 and IL-6 are produced at high levels by tumor-associated DCs (not shown), which is supported by independent reports[36]. These results therefore point to a scenario in which ovarian cancer cells as well as tumor-infiltrating leukocytes secrete multiple factors that induce the expression of CD277 on infiltrating APCs, which would impair the function of tumor-reactive T cells. As we have published, multiple immunosuppressive mechanisms are orchestrated by the abundant regulatory DC population found in solid ovarian tumors[8, 13, 16-18]. The inhibition of T cell-mediated anti-tumor immune responses by immunosuppressive CD277 may be therefore a common mechanism of immune evasion in ovarian cancer patients, and perhaps in other tumors. Importantly, the extracellular domain of BTN3A3 shares 97% sequence identity with CD277, suggesting identical inhibitory functions. In addition, given the redundant activities of other BTNs from different sub-families, multiple BTNs could converge in the tumor microenvironment to inhibit anti-tumor adaptive immune responses and create immune privilege. BTNs can therefore be essential for understanding potential immune evasion in cancer and represent crucial immunotherapeutic targets.

## METHODS

### Ethics statement

Investigation has been conducted in accordance with the ethical standards and according to the Declaration of Helsinki and according to national and international guidelines and has been approved by the author's institutional review board.

### Tissues, cell lines and treatments

Stage III/IV human ovarian cancer tissues (n=30) were obtained through Research Pathology at Dartmouth Medical School, Lebanon, NH, after institutional approval. Single-cell suspensions were generated by gently forcing minced fresh specimens through a 70-μm pore mesh and subjected to Ficoll gradient centrifugation. Human ovarian cancer cell lines SKOV3, Ovcar60 and A2008 were grown to 80% confluency in DMEM medium containing 10% FBS prior to RNA isolation.

Peripheral blood lymphocytes were obtained by leukapheresis/elutriation and Miltenyi bead–purified, as described[5].

Monocyte-derived dendritic cells (MoDCs) were generated by incubating magnetically purified CD14+ cells for 7 days with granulocyte-macrophage colony stimulating factor (20 ng/mL; PeproTech,) and IL-4 (50 ng/mL; R&D Systems). MoDCs were then stimulated for 3 days with IL-10 (200 ng/ml), IL-6 (10 ng/ml), CCL3 (50 ng/ml), VEGF (50 ng/ml), PLGF-1 (50 ng/ml) or filter-sterilized human ovarian cancer ascites at a 1:1 ratio.

Immunohistochemistry was performed using a purified anti-CD277 antibody (clone eBioBT3.1, eBioscience) and the ABC kit (Chemicon International, Temecula, CA), as reported[3, 13, 18]. Horse serum (1/10 dilution) was used as a negative control.

Artificial antigen presenting cells (K32) were generated by stably expressing human CD32 on the surface of K562 cells, as previously described[29, 30]. CD277 was PCR amplified from human spleen cDNA using primers BTN-Fw (5' GTT GGG ACT CAA AGG TGA AGA C 3') and BTN-Rev (5' TGT CTC TAG GGA ATG ATC AGC A 3'). Expression of CD277 on K32 cells was achieved using the MSCV Retroviral Expression System (Clontech), following the recommendations of the manufacturer.

For T cell proliferation experiments, K32 cells expressing or not CD277 were γ-irradiated (100 Gy), washed twice with 1X PBS, and loaded with anti-CD3 alone (100 ng/ml, clone OKT3; eBioscience) or anti-CD3 (25 ng/ml) plus anti-CD28 (100 ng/ml, clone 15E8; Chemicon International) antibodies at room temperature for 10 min. T cells were CFSE-labeled and cocultured with loaded K32 cells at a 10:1 ratio. Proliferation of stimulated T cells was determined 5 days later by FACS on the basis of CFSE dilution.

### RNA isolation, cDNA generation and Real Time PCR

In all cases, total RNA was extracted using Trizol (Invitrogen) and total cDNA was generated using SuperScript III Reverse Transcriptase (Invitrogen) and random primers, following the recommendations of the manufacturer. CD277 mRNA levels were quantified by Real Time PCR using TaqMan assays (Applied Biosystems) and primers qBTN-F (5' GGA GGG TGT ATC CTG TAC CAT CA 3'), qBTN-R (5' AAG AAG CAG CAG CAA GAC AGG 3') and the internal probe pBTN (5' CCT GGA AAA GAC AGC CAG CAT 3'). In all experiments CD277 expression was normalized among samples by quantifying the messenger levels of GAPDH using primers GAPDH-F (5' CCT GCA CCA CCA ACT GCT TA 3'), GAPDH-R (5' AGT GAT GGC ATG GAC TGT GGT 3') and the internal probe pGAPDH (5' CCT GGC CAA GGT CAT CCA TGA CAA C 3')

### Flow cytometry, Bio-Plex and Immunoblotting

Flow cytometry was performed on a FACSCanto (BD Biosciences, San Jose, CA). Cell populations were sorted from human tumor single-cell suspensions using a FACSAria sorter (BD Biosciences). Anti-human antibodies were specific for CD45 (HI30), DEC205 (MG38), CD3 (UCHT1), CD4 (OKT4), CD11b (ICRF44), CD14 (M5E2) all from BD Biosciences; CD277 (20.1, BT3.1) and CD25 (BC96) antibodies were from eBioscience.

Cytokines in supernatants from stimulated T cells were quantified in a Bio-Plex assay (Bio-Rad, Hercules, CA) using the Human-27-Plex panel. Plates were read in a Bio-Plex Array Reader (Bio-Rad).

Intracellular expression of Annexin V was determined using the Annexin V-FITC Apoptosis Detection Kit (BD Biosciences), following manufacturer's instructions.

To determine c-Flip expression, pellets from CD277-exposed T cells were resuspended in 30 μl Laemmli buffer, boiled for 5 min, loaded onto a 15% sodium dodecyl sulfate–polyacrylamide electrophoresis gel, transferred to a nitrocellulose membrane, blocked, and incubated with an anti-human c-Flip antibody (C-terminus; eBioscence). Immunoreactive bands were developed using horseradish peroxidase–conjugated secondary antibodies (Bio-Rad) and chemiluminescent substrates (Pierce Chemical, Rockford, IL). In all cases, membranes were re-probed with an anti-GAPDH antibody (BioLegend) as loading control.

### Soluble proteins and binding assays

The extracellular portion of BTN3A1 (aa 30-254) was PCR amplified from human spleen cDNA using primers eBTN-F (5' aag ctt CAG TTT TC TGT GCT TGG AC 3') and eBTN-R (5' gaa ttc TCT GGG CGC TCC TGA AGA AG 3'), wherein the lower case nucleotides represent HindIII and EcoRI sites, respectively. The amplified product was subcloned in frame into the expression vector pSecTag2B (Invitrogen) following the recommendations of the manufacturer, to generate a secreted protein containing the BTN3A1 extracelullar portion fused to a C-terminal Myc epitope and a poly-histidine tag. HEK293 cells were transfected with the resulting vector and secreted CD277 was purified from culture supernatants 3 days later using the His GraviTrap system (GE Healthcare). To generate a control extracelullar protein (iProt), HEK293 cells were transfected with empty pSecTag2B, which generates an irrelevant His-tagged polypeptide containing a C-terminal Myc epitope. For binding assays, resting or CD3/CD28 bead-activated T cells were incubated with the tagged proteins (0.5 ug/ml) at room temperature for 30 min. Cells were washed stringently and protein binding was assessed by secondary incubation with a FITC-labeled anti-Myc antibody (9E10; Sigma), followed by FACS analysis.

### Prediction of structure and statistical analysis

The structure of the extracellular domains of B7-H4 and CD277 were modeled using PD-L1 with Protein Homology/analog Y Recognition Engine (Phyre)[37]. Predicted structures were overlapped using Rasmol (http://rasmol.org). Phylogenetic analysis of members of the B7 family was performed with ClustalW[38].

Differences between the means of experimental groups were analyzed with the Mann-Whitney test using the GraphPad Prism 4.0 software.

## Abbrevation Used

aAPC: artificial Antigen-Presenting Cell
BTN: Butyrophilin
MDSC: Myeloid-Derived Supressor Cell
VLC: Vascular Leukocyte
DC: Dendritic Cell
VEGF: Vascular Endothelial Growth Factor A
PlGF: Placental Growth Factor
CCL3: C-C motif chemokine 3
